# Affectionate Interactions of Cats with Children Having Autism Spectrum Disorder

**DOI:** 10.3389/fvets.2018.00039

**Published:** 2018-03-12

**Authors:** Lynette A. Hart, Abigail P. Thigpen, Neil H. Willits, Leslie A. Lyons, Irva Hertz-Picciotto, Benjamin L. Hart

**Affiliations:** ^1^Department of Population Health and Reproduction, School of Veterinary Medicine, University of California-Davis, Davis, CA, United States; ^2^Department of Statistics, University of California-Davis, Davis, CA, United States; ^3^Department of Veterinary Medicine and Surgery, College of Veterinary Medicine, University of Missouri, Columbia, SC, United States; ^4^Medical Investigations of Neurodevelopmental Disorders (M.I.N.D.) Institute, University of California-Davis, Davis, CA, United States; ^5^Department of Anatomy, Physiology and Cell Biology, School of Veterinary Medicine, University of California-Davis, Davis, CA, United States

**Keywords:** aggressive behavior of cats, affectionate behavior of cats, autism, autism spectrum disorder, cats and children, anthrozoology, human–animal interaction

## Abstract

Mental and physical benefits of dogs have been reported for adults and children with special needs, but less is known about benefits of cats for children. A cat that can be held by a child could provide important therapeutic companionship for children with severe or less severe autism spectrum disorder (ASD) who otherwise may lack prosocial behaviors. Because relatively little is known about the behavior of cats around children, we conducted this study. Phase 1 gathered web-survey data from families having an adult cat and a child with ASD (*n* = 64). In Phase 2, there were direct telephone interviews of parents having a child with severe ASD (*n* = 16) or less severe ASD (*n* = 11), or typical development (*n* = 17). From the Phase 1 web survey of families with ASD children (full range of severities), affectionate interactions of the cats with children were common. Most parents with ASD children volunteered positive comments regarding the cat, such as calming the child, being a soothing protector or a guardian. In the interviews in Phase 2, for all three groups, most parents characterized cats as at least moderately affectionate toward the child. However, cats living with severe ASD children were reported to exhibit less affection than those living with typically developing children or children with less severe ASD. A minority of cats in each group showed some aggression to the specified child; this was not elevated with ASD children. Responses suggested that the cats adopted as kittens were more affectionate and less aggressive to all categories of children than those adopted as adults. Overall, participants reported that ASD children’s behaviors indicated that they valued the relationship with the cat, similar to typically developing children, pointing to the importance and potential usefulness of selecting affectionate and compatible cats for ASD children.

## Introduction

Many families with children have dogs and/or cats as pets, and much has been studied in recent decades about the value of pets for the emotional and physical development of children in the typical family (1). Introducing a pet—dog, cat, or hamster—to children with autism spectrum disorder (ASD) was associated with an increase in the children’s prosocial behaviors, as compared with children lacking a pet (2). Canine companions have been particularly studied for the mental and physical benefits of dogs for children with ASD (3). The term, ASD, is inclusive and now encompasses the range of mild-to-severe autistic impairments (4).

Service dogs have particular utility in assuring the safety of the child, giving the family respite, taking the social focus off the child, adding stature to the family and assuring safety by keeping the child from bolting and running away (5). Service dogs were shown to decrease cortisol secretion upon awakening in children with ASD, and parents reported improvements in behavior of the child when having a service dog (6). Not surprisingly, the use of dogs with autistic children is an expanding role for service dogs, with the numbers of dogs placed in families with an autistic child increasing among facilities accredited with Assistance Dogs International (ADI) and also among non-accredited facilities in the U.S. (7).

Although dogs have the capacity to perform useful tasks and are more interactive with people than cats, they require more attention and care, and some parents reportedly find their ASD child is more compatible with a cat, or that a dog simply would not be a feasible companion for their child (2). In some other contexts, cats have been found to be a better lifestyle fit. For example, people with AIDS (acquired immune deficiency syndrome), who have cats are reported to enjoy their cats, find them comforting, and are spared concerns of many dog owners who are expected to fulfill their dogs’ needs for exercise and being taken outdoors for elimination (8). Middle-aged women who were caregivers at home for an elderly family member with Alzheimer’s disease reported that, for them, a cat was a more compatible and satisfying pet than a dog (9). Having had cats was even found in one study to be associated with fewer deaths from heart disease than having had a dog or no pets (10).

We hypothesized that a relaxed cat that can be held and/or carried about could be a therapeutic companion for a child with ASD, perhaps offering ongoing calming comfort supplementing that from a parent or other human family member, and also facilitating social behavior by the child.

The goal of this study was to obtain background information about the interactions of cats with children in small samples of families who have a cat and a child with ASD. These children presumably could benefit emotionally, and perhaps cognitively, from contact warmth and affection that might be supplied by an appropriate pet cat. A cat that “belonged” to the affected child, and was affectionate and liked to be held, could offer a positive relationship for the child and supplement the parents’ affection and be emotionally beneficial, or perhaps even bridge to other social interactions for the child. Given the nature of ASD, dogs may be less appropriate for providing ongoing contact affection to some children. It could even be possible that an autistic child might be educated not only to interact appropriately but also to partially care for the cat and even verbally communicate with the cat (if demonstrated by the parents).

It is relevant to point out that cats vary a great deal in affection and aggressive behavioral predispositions toward family members. This variability is also evident when comparing purebred cats (11). Selecting a purebred that is genetically predisposed to be affectionate and comforting could play a role in the assessment of which cats would be most likely to be best for a child with ASD. An extensive study on cat breeds revealed that the most affectionate, socially outgoing, and least aggressive, breed is the Ragdoll. While not approaching the Ragdoll in the absence of aggression, the popular domestic shorthair also was rated as very affectionate. In terms of sex, neutered males were rated as being more affectionate than females. Although genetics and gender are important, so also are the manner in which the cat is reared and managed and the ways in which humans behave toward the cat.

Expecting that a cat would be affectionate with a child may pose particular challenges, since cats were found by Mertens (12) to prefer adults to young children, in terms of approaches and duration of proximity. Cats in families preferred adult women, with whom they reportedly had their most reciprocal relationships. These findings raise a possibility that predicting the interactions of cats with children may be more challenging.

In this study, Phase 1 gathered data in a web-based survey on the nature of cat–child interactions in families with an ASD child. Most behavioral questions pertained to the extent to which the cats’ characteristics were: very affectionate, or at least moderately affectionate, low in fearfulness, and relatively non-aggressive with the children. Two questions concerned the responses of the children to the cats. We then explored similar questions in Phase 2, by virtue of direct structured telephone interviews of adults in families with children who have ASD, comparing cats’ interactions with children with confirmed diagnoses of severe ASD or less severe ASD as well as a sample of families with typically developing children.

## Materials and Methods

### General Methods for the Two Studies

In addition to demographic information on family members and pets in the household, parents provided behavioral ratings for the specified cat and specified child, as well as other members of the household, on a five-point scale. The cat’s affectionate interactions were categorized as: very affectionate (loved being held or carried around); affectionate (mostly liked being held or carried); moderately affectionate (liked some degree of being held or carried); relatively non-affectionate (preferred not being held or carried); and non-affectionate (did not like being held or carried). The aggressive interactions were categorized as: very aggressive (family members limited exposure to cat); quite aggressive (family members had to be on alert around cat); moderately aggressive (sometimes acted up when held too much); relatively non-aggressive (occasionally would get irritated); and non-aggressive (regardless of how interacted with).

Fearfulness, toward visitors, was categorized as: very fearful (runs away and stays hidden); fearful (runs away, eventually comes out); moderately fearful (may or may not hide depending on who is present); relatively non-fearful (greets most, but not all, visitors); and non-fearful.

Reponses of the specified child toward the specified cat were categorized as: indifferent to cat; fearful of cat; sometimes likes to hold or sit with cat; moderately responsive (holds or sits with cat half of the time when the cat is around); usually likes to hold and pet cat when around; always seems to want to hold, pet, snuggle, and sleep with cat; and other (explanation could be provided).

### Institutional Review Approval Board

Approval was obtained from the University of California, Davis, Institutional Review Board as Protocols #201018447-1 and #284059-2.

### Phase 1. Web Survey of Parents Having both a Child Who Had Been Diagnosed with ASD and a Cat

To clarify the characteristics of a cat that make it a desirable companion for a young child, we designed a 39-item web-based survey in SurveyMonkey directed toward families having an adult cat and a child diagnosed with ASD. The survey included the stated requirement that participants had to have a child within the age range of 3–12 years and a cat at least 1 year of age. We distributed the web-link and solicited participation *via* listservs and groups serving families that have children with ASD. The survey did not require details on the child’s diagnosis. This survey was available for responding May 2013 through June 2014.

The UC Davis Institutional Review Board (IRB) approved a written informed consent waiver because of the anonymity of participating parents since no identifying information was requested in the survey. Participants were informed that they were participating in a research survey, and by completing the survey, they were consenting to the use of their responses in a study analysis. Participants were required to be 18 years of age or older to submit the survey.

Among the 88 respondents to this web survey, 64 met the following inclusion criteria: responding adults having at least one child aged 3–12 years diagnosed with ASD (the specified child); having in the household at least one cat 1 year of age or older (the specified cat); completing the 39 questions of the survey; and residing in North America. The sociodemographic information gathered included: gender and age of the specified child; gender, breed, age and source of the specified cat; household information on adults and other children in the household; and information on numbers of dogs and other cats in the household. Behavioral questions regarding the specified cat addressed: sleeping location, usual daily time spent with the child, and ratings of the cat on affection, aggression, and fearfulness, playfulness, and friendliness with visitors. Behavioral questions regarding the specified child addressed information on the specified cat’s interactions with the child, and the child’s level of interest and responses to the cat: the child being fearful of the cat; indifferent to the cat’s affection; liking to sit with or hold the cat; being moderately responsive to the cat; usually loving to hold and pet the cat; or always wanting to hold, pet, snuggle, and sleep with the cat; or other (to be specified). Types of interactions for the child interacting with the cat that could be selected by respondents included multiple options: frequently talking to the cat; frequently attempting to read to the cat; frequently attempting to play with the cat; liking to feed or give treats to the cat; or none of the above. A final question invited respondents to briefly comment on their experiences with young children interacting with cats.

### Phase 2. Direct Interviews of Parents of a Child Diagnosed with Severe ASD, Less Severe ASD, or Typical Development

Drawing from a database of parents’ names provided by the University of California, Davis, School of Medicine, M.I.N.D. Institute CHARGE (Childhood Autism Risks from Genetics and Environment) study (PI: IHP), we acquired 557 potential participants’ names and mailing addresses. The database included families with a child diagnosed with: severe ASD; less severe ASD, often referred to as Asperger’s syndrome; delayed development; typical development; and a non-specified diagnosis. These families had cats and had previously indicated a willingness to be contacted. Inclusion criteria included that the child be 5–12 years of age.

All 557 potential participants were sent a packet including a letter with instructions on how to participate in the study, consent forms, the participant’s bill of rights, and an IRB-approved brief description of the study. Participants were invited to reply through the mail with the signed consent form.

We presumably reached 515 parents with a mailed invitation to participate (mailed packets not returned). Sixty-four of these potential participants replied through the mail, volunteering to participate in a telephone interview. Among those who still had a cat and could be reached by telephone, 48 phone interviews with the responsible adult were completed and met the inclusion criteria. The 48 interviews were conducted in January 2012–June 2014. The single interviewer who conducted all interviews did not know the category of diagnosis of the child when interviewing the parent.

The primary emphasis was to characterize cats’ behaviors with the children and compare cats’ interactions with children having ASD, less severe ASD, or typical development. The interview questions were drawn directly from the web survey, but the interviews permitted more extensive responses than the multiple choices possible in the web survey. A parent provided ratings by telephone of the cat–children interactions according to degrees of affectionate, aggressive, and fearful interactions, playfulness with the children, and the extent to which children liked holding and interacting with the cats. After the 48 phone interviews were completed and the responses were scored and the participants given unique identifiers, the diagnoses of children, among the five types listed above, were provided. The data that had been collected pertained to 16 children diagnosed with severe ASD, 11 with less severe ASD, 17 designated as being typical, and 3 with delayed development, as well as 1 child with incomplete diagnoses. Included here are data on 44 children–cat pairs, for children with severe ASD, mild ASD, or diagnosed as typically developing.

### Statistical Analyses

Data of the two studies are reported using descriptive statistics, using medians, and the results of chi-square or Fisher exact tests for significance. For the survey data from Phase 1, 12 responses were identified as reflecting the quality of interactions between the autistic child and the cat. These included: cat sleeping in the child’s room; cat sleeping on the child’s bed; cat being within the child’s arm’s reach on the bed; time cat spent with the specified child; cat’s affection toward the child; and nature of the child’s reactions to the cat, including talking to the cat, reading to the cat, playing with the cat, giving food to the cat; and the cat’s aggression toward the child; and cat’s playfulness with the child. A principal component analysis (PCA) was run on these variables, and the first principal component explained 31% of the variability in the responses. In this analysis, three of the 64 subjects were excluded, due to incomplete values for one or more of the responses. The factor loadings for the first factor were all positive except for “aggression toward child,” which was negative. The first factor was used as a dependent variable in running several one-way ANOVA models, looking for systematic differences with respect to the child’s gender, the source where the cat was acquired, the cat’s gender, the cat’s breed (unknown, DSH, DLH, and “purebred”), and whether the cat was gonadally intact or not. The residual errors from these analyses were checked for normality using Wilk Shapiro tests, all of which indicated a close agreement with a normal distribution (*W* > 0.95). The first factor was also used as the dependent variable in a regression tree analysis (CART) that used a broader array of explanatory variables. All analyses were run using SAS, version 9.4, except for the regression trees, which were run using R statistical software and the rpart command.

## Results

### Phase 1. Web Survey of Parents with a Child at Least 3–12 years of Age Diagnosed with ASD

#### Family Demographics and Ages of Children and Cats

The survey was of 64 families with an ASD child and a specified cat, so as to characterize the relationships of the child and the family with the specified cat as described by an adult family member. When families had multiple cats, the parent responded concerning a specified cat that was most interactive with the child. Most children resided in households that included several family members and animals.

Only slightly over half of the families had other children in the home. Concerning the age ranges of all children in the household (including the specified child), only 17% of households had teenagers 13–19 years of age, with higher proportions of households with children of other ages: 44% with children 10–12 years, 61% with children 6–9 years, and 38% with children 3–5 years (Table 1). The specified child with ASD was a boy 72% of the time with the median age range of 6–9 years. Most families had multi-pet households, with 38% having one or more dogs. A majority of households, 52%, had multiple cats over the age of 1 year, so, most homes offered the child a choice between at least two cats.

**Table 1:** General descriptive information of households having a severe or less severe autism spectrum disorder (ASD) child and a specified cat, Phase 1.

		Number of respondents (%)
Living in the house	Other children present	37/64 (58%)
	Dog(s) present	24/64 (38%)
	1 cat	30/64 (47%)
	2 cats	16/64 (25%)
	3 cats	14/64 (22%)
	4 cats	3/64 (5%)
	More than 4 cats	1/64 (2%)

Specified cat	1–3 years old	31/64 (48%)
	4–6 years old	14/64 (22%)
	7–10 years old	13/64 (20%)
	Over 10 years old	6/64 (9%)
	Generic shorthair	33/64 (52%)
	Generic longhair	8/64 (13%)
	Purebred	13/64 (20%)
	Sleeps outdoors	1/64 (2%)
	Sleeps indoors	63/64 (98%)

Of the specified cats that interacted with the specified child the most, the median age range was 4–6 years. Male neutered (42%) and female spayed (42%) accounted for most specified cats. A majority of the cats (52%) were domestic shorthair.

#### The Cat’s General Behavior with the ASD Child and Typical Children

Of the specified cats interacting with the ASD child, 78% were at least somewhat affectionate, with 30% rated as very affectionate (Table 2). The latter rating was described as the cat loving being held and carried around by the child. In contrast, among all cats, 22% were very affectionate to adults, 5% toward children ages 10–12 years, 9% toward children ages 6–9 years, and 9% toward children ages 3–5 years.

**Table 2:** Behaviors of cats with severe or less severe autism spectrum disorder (ASD) children in Phase 1, and ASD children’s behaviors with cats.

		Number of respondents (%)
Affection of cat (decreasing degrees are inclusive)	Very affectionate	19/64 (30%)
	At least mostly affectionate	31/64 (48%)
	At least moderately affectionate	50/64 (78%)

Child’s interaction with cat	Child at least usually wants to hold, pet, snuggle, and sleep with cat	35/64 (55%)
	Child always wants to hold, pet, snuggle, and sleep with cat	22/64 (34%)
	Child frequently talks or reads with cat	41/64 (64%)

Of the 19 specified cats that were very affectionate toward the specified child, not all were affectionate toward adults, as indicated by only eight of these cats (42%) being very affectionate toward adults (*p* < 0.05). The same was true for other children in the family (*p* < 0.05). Thus, these very affectionate cats were more affectionate to the specified child than to adults or other children in the family. Among the 19 cats that were very affectionate to the specified child, five (26%) were over 6 years of age; 31% of the remaining less affectionate cats were over 6 years of age (ns).

In addition, all specified cats were relatively low in aggression, with 47% never being aggressive to the specified child. And 47% of cats also were never aggressive to adults, but only 25% of specified cats were never aggressive to another child in the household. Mirroring the affection results, this leads to the assumption that these cats were more likely to be attached, affectionate, and non-aggressive to the ASD child and often preferred the specified child rather than adults or other children in the household.

#### Cat’s Behavior Affecting the Child–Cat Relationship

Most of the ASD children (55%) always or usually wanted to hold, pet, snuggle, or sleep with the cat (Table 2). However, the median range of time these children were reported to actually spend per day with the cat was just 1–2 h. Only 25% of these cats slept in the child’s bedroom. Despite the child’s strong interest in the cat, most of the hours of the day most cats spent much of their time apart from the specified child.

As described in the Statistical Methods, a PCA was run on all responses that pertained to the quality or depth of the relationship between the autistic child and the specified cat. The child’s gender and the cat’s gender, intact status, and breed first were found to be insignificant factors. The first principal component summarized this information, with positive factor loadings for all positive cat/child interactions, except for “aggression toward child,” which had a negative factor loading. Thus, high values of the first principal component indicated a positive/deep relationship, and low values indicated a poor/shallow relationship.

**Figure 1:**
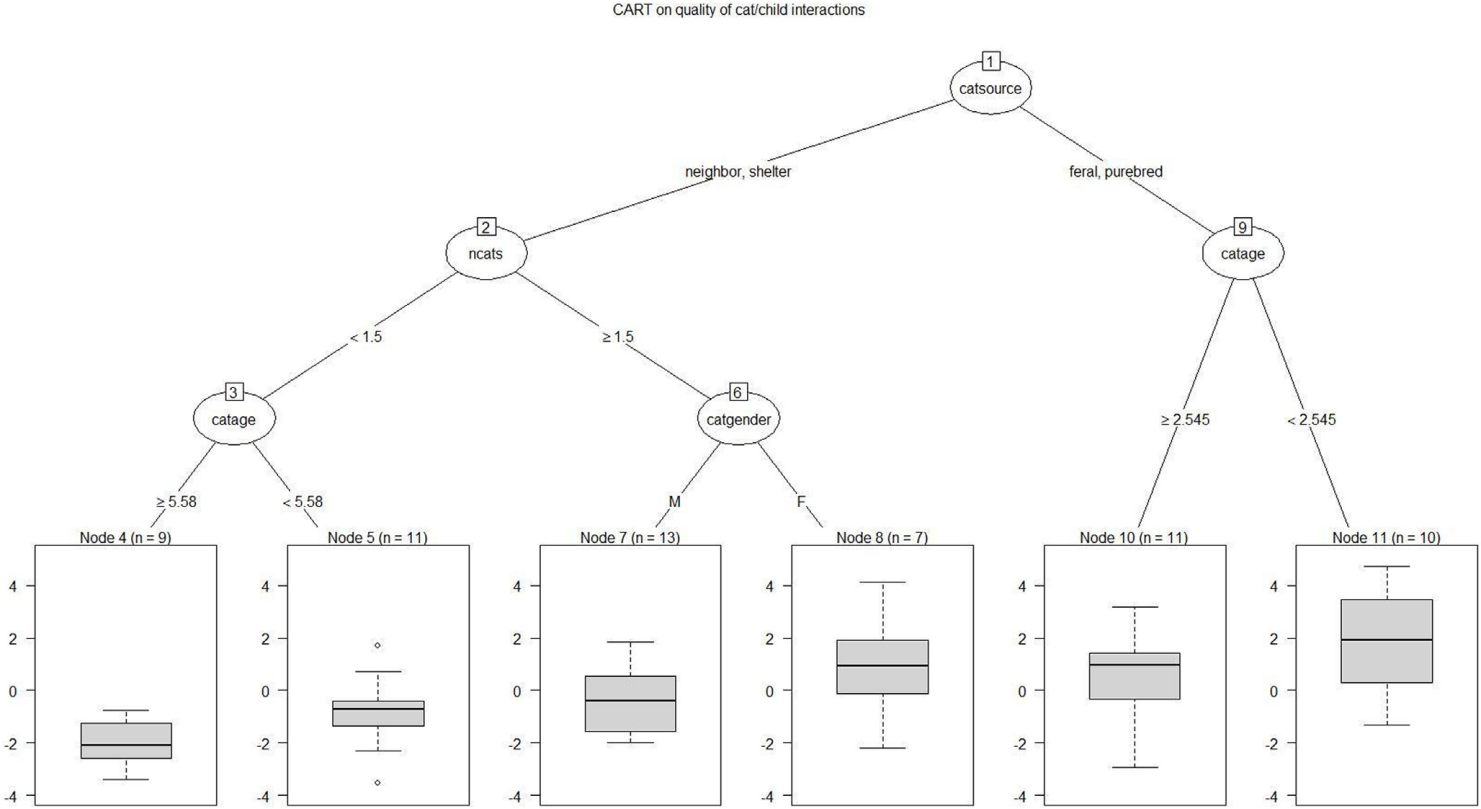
Regression tree CART analysis depicting variables affecting the quality of child/cat interactions. The highest quality child/cat interactions appear on the right side of the graph. The primary node (the first split) depended on the source of the specified cat: cats from a shelter or from neighborhood breeders had the lowest quality interactions with the autistic child, and cats adopted as ferals or from a purebred breeder had the highest quality interactions. Among feral/purebred cats, the highest quality interactions were for younger cats. Among neighborhood/shelter cats, the least successful interactions were for single cat households, and beyond that, older cats.

A regression tree was run on the value of the first principal component, using a series of demographic variables as potential predictors. The goal of this analysis is to define predictors and threshold values that distinguish between low and high values of the response. The results of this analysis are presented in Figure 1, where the nodes with the highest quality child/cat interactions appear on the right of the graph, while the nodes with lowest quality interactions appear on the left. The primary node (the first split) depended on the source of the specified cat, with cats from a shelter or from neighborhood breeders having the lowest quality interactions with the autistic child, and cats adopted as ferals (*n* = 19) or from a purebred breeder (*n* = 2) having the highest quality interactions. Among the feral/purebred cats, the highest quality interactions were for younger cats (less than 2.5 years of age). While data were not gathered on the cat’s age at adoption, among the 64 respondents, 9 of the feral cat adopters and only one of the neighborhood adopters volunteered that they had adopted the cat as a kitten; none of the shelter adopters mentioned acquiring a kitten, nor did the two purebred adopters where adopting a kitten would be likely. Beyond that, within the neighborhood/shelter group, the least successful interactions were for single cat households, and beyond that, older cats (age 5.58 or greater). Among multiple cat households in the neighborhood/shelter group, female cats had somewhat better quality relationships (not significant) with the specified child than male cats.

Of the 64 respondents, 52 parents volunteered comments regarding interaction of the cat and the child: 40 comments were positive, three neutral, and nine negative. Comments volunteered from 19 parents characterized behavior of the cat in being a calming, loving, soothing protector, bonded friend, or guardian for the child. The child’s feelings for the cat often were described as: loving, enamored, or bonded. Mood regulation of the child was mentioned as an effect of the cat. Less-positive descriptions pertaining to behavior of the child or cat included: “My son does not interact at all with cats or dogs; he just ignores them”; “In the past, when the child was young, the cat did not like the hyper behavior of the child and would avoid most interaction, but as the child has gotten older, the cat will seek out the child.”

Parents described the special role of the cat for the child with the following sample of quotations: “When the specified child is upset, the cat will sit by or in the lap of the specified child.” “The only time we get to hear our autistic child spontaneously speak is when he is interacting with this cat.” “He is non-verbal and doesn’t sign for much, but he does sign for his kitty numerous times each day!” “My son just likes to look at the cats and talk to them.” “He will sit and read to the cats although you can’t understand what he is saying.” “The specified cat helped my child say new words.”

One quotation regarding an autistic child attached to two cats was quite noteworthy: “Our Tonkinese are amazing with my autistic daughter. They understand her moods and needs. They respond to her so incredibly. When she does not bond with humans’ touch, she does with her cats. They bring her back to me. They are the bridge I need so that I can enjoy my daughter more. When she has them on her lap, I can hold her hand. They serve as a buffer, a calming energy. They know their role. They cry to be let in her room. They choose her lap over mine when hers becomes available. They are truly amazing!”

### Phase 2. Interview Results

#### Family Demographics, Children’s Diagnoses, and Cats’ Descriptions

These parents were contacted after some time had passed since they had initially enrolled with the University of California, Davis, Mind Institute. Interviews meeting the inclusion criteria included: 44 respondents with cats, whose children had diagnoses of severe ASD (*n* = 16), less severe ASD (*n* = 11), or typical development (*n* = 17). The specific cats in each group were: for ASD children, 5 neutered males and 11 spayed females, including 11 domestic shorthair or longhair cats and 5 other breeds; for less severe ASD children, 7 neutered males and 4 spayed females, including 7 domestic shorthair or longhair cats and 4 other breeds; and for typical children, 9 neutered males and 8 spayed females, including 12 shorthair or longhair cats and 5 other breeds.

All of these households had at least two adults except two typically developing child’s and one less severe ASD child’s households. Most families had multiple children, excepting one family in the typical group, four families in the less severe ASD groups, and five families in the ASD group. Families in the typical group were significantly more likely to have additional children in the home, as compared with ASD or less severe ASD groups (Table 3). All families of typical children and 91% of families of less severe ASD children had dogs. Most families (87.5%) of ASD children also had dogs and half of these had more than one dog. About half of the families had more than one cat; typical, 48%; mild ASD, 45%; and ASD, 44%.

**Table 3:** General descriptive information of households with a child and a specified cat, based on child’s diagnosis, Phase 2.

		Number of respondents (%)		

		Autism spectrum disorder (ASD)	Less severe ASD	Typical
Living in the house	Other children present^a^	5/16 (38%)	7/11 (64%)	16/17 (94%)
	Dog(s) present	14/16 (88%)	10/11 (91%)	17/17 (100%)
	1 cat	9/16 (56%)	6/11 (55%)	9/17 (53%)
	2 cats	5/16 (31%)	4/11 (36%)	7/17 (41%)
	3 cats	2/16 (13%)	0	0
	4 cats	0	1/11 (9%)	0
	More than 4 cats	0	0	1/17 (6%)

Specified cat	1–3 years old	1/16 (6%)	4/11 (36%)	5/17 (29%)
	4–6 years old	5/16 (31%)	1/11 (9%)	1/17 (6%)
	7–10 years old	5/16 (31%)	2/11 (18%)	3/17 (18%)
	Over 10 years old	4/16 (25%)	4/11 (36%)	6/17 (35%)
	Generic shorthair	9/16 (56%)	6/11 (55%)	11/17 (65%)
	Generic longhair	2/16 (13%)	1/11 (9%)	1/17 (6%)
	Purebred	2/16 (13%)	4/11 (36%)	3/17 (18%)
	Sleeps outdoors	2/16 (13%)	1/11 (9%)	3/17 (18%)
	Sleeps indoors	11/16 (69%)	10/11 (91%)	11/17 (65%)

^a^Fisher test: *p < 0.0004.

#### The Cat’s General Behavior with the Child

**Figure 2:**
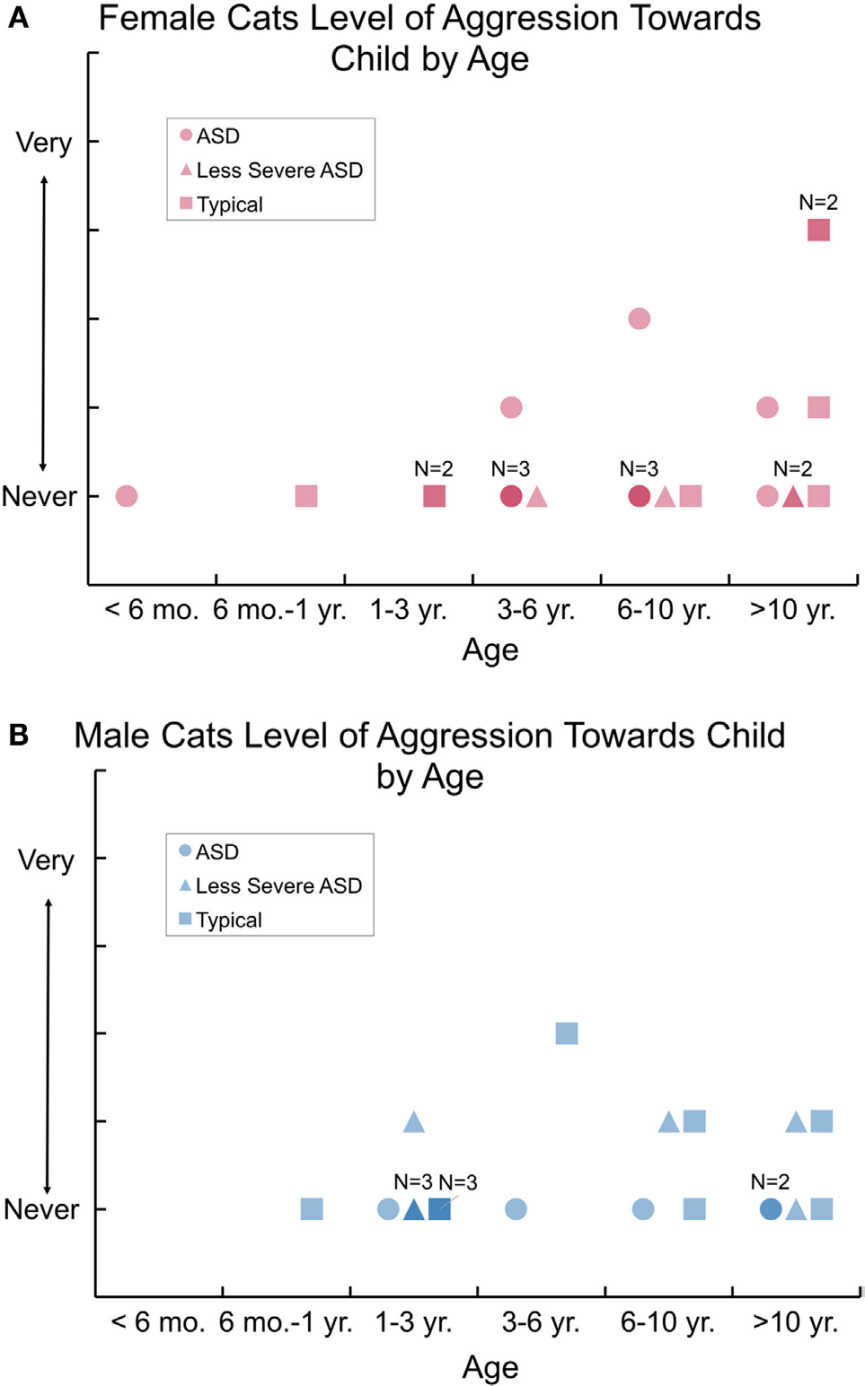
The level of aggression of each female **(A)** and male neutered cat **(B)** cat plotted by the cat’s age and group. Aggression to severe autism spectrum disorder (ASD) children was no worse than with less severe ASD or typical children.

Results of interviews are shown in Table 4, rating the cat–child interactions on the scales for the cat’s aggression, affection, and playfulness, as well as the child liking to hold the cat. A minority of cats showed some aggression with the specified child: 19, 27, and 35% for cats of ASD, less severe ASD, and typical children, respectively. Among all cats, 71% of males and 74% of females were scored as never aggressive with the specified child. Overall, the highest aggression scores of cats with the specified child were for two female cats of typical children (“aggressive enough that we have to be alert”), and one female cat of an ASD child and another female cat of a typical child scoring as “moderately non-aggressive.” Figures 2A,B for male and female cats shows the level of aggression of each cat plotted by the cat’s age and group, showing that aggression to ASD children was no worse than with less severe ASD or typical children. Nonetheless, as shown in Table 4, when also comparing the aggressive behaviors for cats of severe ASD children with adults and all children or cats in the same household (63%), the specified cats’ levels of aggression were significantly heightened above that shown only to the specified child (19%): *p* < 0.012.

**Table 4:** Behaviors of cats with children in Phase 2 diagnosed as autism spectrum disorder (ASD), less severe ASD, or developing typically, and children’s behaviors with cats.

		Number of respondents (%)		
		ASD	Less severe ASD	Typical
Affection of cat (decreasing degrees are inclusive)	Very affectionate*	3/16 (19%)	7/11 (64%)	9/17 (53%)
	At least quite affectionate	6/16 (38%)	8/11 (73%)	12/17 (71%)
	At least moderately affectionate	11/16 (69%)	9/11 (82%)	16/17 (94%)

Playfulness of cat	At least moderately playful**	6/16 (38%)	8/11 (73%)	12/17 (71%)

Aggressiveness of cat	Any aggression toward specified child	3/16 (19%)	3/11 (27%)	6/17 (35%)
	Any aggression toward specified child, adults, other children, and/or other cats	10/16 (63%)	4/11 (36%)	11/17 (65%)

Fearfulness of cat	Very fearful toward visiting children and/or adults	7/16 (44%)	3/11 (27%)	3/17 (18%)

Child’s interaction with cat	Child at least usually wants to hold, pet, snuggle, and sleep with cat	11/16 (69%)	6/11 (55%)	10/17 (59%)
	Child always wants to hold, pet, snuggle, and sleep with cat	4/16 (25%)	4/11 (36%)	6/17 (35%)
	Child frequently talks or reads with cat***	8/16 (50%)	10/11 (91%)	15/17 (88%)

Fisher tests: *p < 0.040; **trend p < 0.093; ***p < 0.019.

A majority of cats in all groups were at least moderately affectionate with the children. Specified cats were significantly less likely to be very affectionate with severe ASD children when compared with the specified cats for the less severe ASD or typically developing children (*p* < 0.040). However, the likelihood of the cat being quite affectionate or moderately affectionate with the child did not differ among groups.

Ratings of each cat’s level of affection to the specific child, adults, and other children in the family were compared. With the 17 typically developing children, one cat was more affectionate to adults, and two more affectionate to the specified child; among the 16 of these children who had siblings, two cats were more affectionate to the specified child than other children in the family. Among the 11 less severe ASD children, three cats were more affectionate with adults and two with the specified child. Among 16 ASD children, five cats were more affectionate with adults than the specified child and one cat was more affectionate with the child than adults.

#### Specified Child’s Reaction to the Specified Cat

Autism spectrum disorder children generally liked holding the family cat (some always wanted to hold, pet, snuggle, and sleep with cat). ASD, less severe ASD, and typical children all liked to hold the cat in about the same proportions (55–69%). The median time range per day the child spent with the cat each day was 30–59 min for typical and ASD children, and 1–2 h for the less severe ASD children.

While there was a range in reports of relationships with the specified cat and autistic children, for a majority of respondents, there was a very favorable relationship between the cat and ASD child, as well as with cats and less severe ASD and typically developing children. Among parents of typically developing children, 9 offered very positive comments. “Cat and child love each other.” “Child likes to talk to our baby about the cat and bring the cat over to the infant to say hi.” “Child loves to carry the cat around.” “Child loves the cat.” “Cat tries to guard the family.” “Fun to watch the children loving to interact with the cat.” Two parents had no comment. Another described the cat and child ignoring each other, and two said the child was slightly fearful of the cat. Two said the children hated and were afraid of unfriendly cats; one usually unfriendly “cat comes into the bedroom at night purring to get petted, child will scream for parents to come get the cat.”

Positive comments from parents of six less severe ASD children included: “Son says the cat definitely improves his quality of life.” “Cat calms down child.” “Child is always looking for the cat.” “Cat follows child throughout the day.” Parents provided two negative comments concerning children who ignored the cat: “Cat and child are fine ignoring each other”; “cat occasionally seems more interested in child than child is in cat.”

Among parents of children diagnosed with ASD, nine offered positive comments. “Cat is a lover, not a fighter, very tolerant.” “Very nurturing cat.” “Child would much rather have a service cat (than a dog).” “She’s the child’s therapy cat.” “Child prides himself in the special bond.” “It was better to get a quiet cat (than a barking dog).” Other comments mentioned the child’s disinterest in the cat (*n* = 3), dislike or fear of the cat (*n* = 2), or the child being a bit rough on the cat (*n* = 1), or had no comment but offered to send a picture. One parent wondered if the lack of bonding at the beginning, when the child was less than 2 years old, accounted for the lack of interaction 8 years later.

### Comparisons from the Two Phases of the Study

Considering results across the two phases and as summarized (Table 5), 88% or more of the cats for all groups in both phases of this study were at least moderately affectionate to adults. A somewhat lesser percentage, 69% or more, were similarly affectionate to children.

**Table 5:** Comparing percentages of cats rated as moderately affectionate to children and adults: Phase 1 web survey of families with a severe or less severe autism spectrum disorder (ASD) child; Phase 2 interviews of parents with child of specified diagnosis.

Cats at least moderately affectionate		
	**Number of respondents (%)**	
**Web survey: Phase 1**	**Adults**	**Specified child**
Severe or less severe ASD child	58/64 (91%)	50/64 (78%)

**Interviews: Phase 2**	**Adults**	**Specified child**
Severe ASD child	14/16 (88%)	11/16 (69%)
Less severe ASD child	10/11 (91%)	9/11 (82%)
Typical child	16/17 (94%)	16/17 (94%)

We found no effect of neuter status or gender related to the aggression or affection shown by the cats. Being the only cat in the house seemed to be a risk factor for heightened aggression and reduced affection by the specified cat; however, in such cases, the child had only one cat available.

Cats in households with an ASD child appeared to be affectionate and minimally aggressive with the ASD child. However, the cat’s level of affection seems higher among cats living with children developing typically as compared with those with severe or less severe ASD.

#### Limitations of the Research

Participants all knew that having a cat was an inclusion criterion of the study. We can assume that this increased participation by people whose families had more positive relationships with their cats. Some families with ASD children may have sought to facilitate positive relationships with pets, perhaps even to selectively acquire a cat for the child, and our survey was designed to explore how well that was working in those families. Families with children may choose to relinquish aggressive cats, especially if they are dealing with an ASD child. No information was gathered on whether a cat had previously been relinquished. The study did not randomly survey about cats in the families. In fact, in multi-cat households, respondents were asked to answer the survey with regard to the cat most interactive with the children. Most families had multiple pets, so, they were optimizing opportunities for the child to connect with an animal. The survey did not ask respondents for the age of adoption of the cats in the families, including the specified cat. While we address the issue in the discussion, in retrospect, this was an important omission.

Recruiting participants from the CHARGE study yielded fewer participants than we had expected. These families already deal with many diagnostics and assessments of their children and face many challenges in providing resources, care, and education for their children. They may have felt that they lacked the time to add on something more. It is possible that those parents who responded were having more positive experiences with their cats than did parents who were non-respondents.

The study did not include an opportunity to conduct direct observations of the interactions of the child and cat; rather, parents provided their perspectives on the behavior of the cat and the relationship of the child and the cat based on their ongoing lives with child and cat. Further, the role of the cat’s breed could not be assessed because of the small numbers of cats.

## Discussion

This study focused primarily on the particular features of cats’ interactions with children having ASD or less severe ASD, and these children’s responses to the cats. Research involving interactions of animals with children having ASD has addressed the animals’ contributions to the social behavior and development of the child, most often in numerous studies with dogs [reviewed in Ref. (13)]. Even guinea pigs (14) and robotic animals have been explored as aids in the social development of children with ASD (15). Other highlighted outcomes studied have included the animal’s contribution to the safety of the child (4), the emotional connection with the child (16), reduction of the child’s problem behaviors, and the child’s cortisol levels upon awakening (6).

Other research has addressed the isolating and stigmatizing experiences for parents who have a child with ASD. Parents often feel disconnected from their autistic child and other family members and are required to engage in extremely vigilant parenting of the child (17). Acquiring a pet dog was found to be associated with improved family functioning and reductions in parenting stress with these children (18). This study on the behaviors of cats with young children focuses on the affectionate behavior that cats demonstrate toward children and that is appreciated by most children, and is calming and comforting to the children.

Most parents of ASD children reported affectionate behavior to the children by the cat. The positive interactions of cats with ASD children revealed that cats can provide an avenue of positive relationships. When there were limitations in the relationships, these usually appeared to be from the cats’ unwillingness to be affectionate rather than the child being disinterested.

It was clear that the specified cats varied considerably in behavior. The study has revealed the importance of having a cat that is inherently low in aggression, socially outgoing, and affectionate as a family companion for a child with ASD or that is typically developing. One such resource that explores purebreds and domestic shorthair and longhair cats in this regard has rated the breeds on scales for these traits (11). A study where adult cat owners rated personality attributes of their cats also suggests the importance of careful pet selection. Six personality dimensions were identified, and one of these, amiability, was strongly correlated with the respondent’s satisfaction with the cat and the bond quality, and the extent to which the cat was not perceived as troublesome (19). Active selection for an affectionate cat could improve the chances of a calming rewarding relationship of cat and child.

In both phases of this study, a minority of cats was reported as sometimes being aggressive with the specified child, adults, other children, or other cats. Importantly, aggression was not heightened with ASD children. Consistent with other studies, a somewhat higher proportion of cats were affectionate with adults than the specified child (Table 5). Yet, most cats were affectionate with the ASD child, a somewhat surprising result given earlier reports of cats more often approaching and spending longer times with adult females rather than other family members (12). Over decades, Turner (20) conducted extensive studies of human–cat interactions, clearly revealing that either the cat or the person can initiate the human–cat interaction, that this affects the subsequent duration of the interaction, and that both parties play very active roles in the interactions and relationship. Further studies using methods similar to those of Turner could clarify the exact nature of the interaction between cats and ASD children.

Many children seek an affectionate relationship with their cats and may benefit from the affection, but their desires are often not fulfilled. Cats that are affectionate to adults, but that are not affectionate to young children, may not tolerate attempts to be held by a child. Some well-known rearing practices of kittens, that would logically predispose cats to being affectionate to young children, are socially habituating (socializing) kittens to several young children and even dogs and other cats. The early weeks of cats’ lives are known to be a sensitive period for inducing friendly, affectionate behavior in cats (21, 22).

When considering placing a cat with a child with ASD, the cat’s welfare is sometimes a concern. We found that in most cases, the cat was spending only an hour a day or less with the child. Appropriately, this means that the cats were able to spend most of the day in various other activities, and, if the child’s behavior with the cat could be problematic, supervising the child’s behavior with the cat would not require extensive time commitments from a parent.

For children known to be consistently kind with the cat, the relationship could offer an at-home brief break for the parents. In the Netherlands, animals are used to provide a short break for children with ASD at care farms (23), but having a calming animal at home offers a more consistent and convenient source of affection. Simply seeing the child being calmer with the cat can be comforting to the parents, as indicated in some of the volunteered comments by parents.

Cats likely to be affectionate may provide rewarding relationships for children with ASD. Most children with diagnosed ASD liked to hold the specified cat (or even always wanted to hold, pet, snuggle, and sleep with cat)—at similar levels as in typically developing children. Concerning the cats, most were at least moderately affectionate toward the ASD child, with almost 20% very affectionate. While the cats generally were affectionate with the ASD children, it was noticeably less than with typically developing children. Importantly, the results revealed that cats showed little aggression with ASD children, and certainly no more than with typical children. It seems that cats in families with an ASD child often provided valuable bonding, attention, and calming affection to the child.

Although the surveys did not ask about the age of the cat at adoption, half of the feral cat adopters voluntarily mentioned acquiring their cats as kittens (whereas adopters from other sources did not); thus, we attribute the positive results for feral and purebred cats to their younger status at adoption, which is consistent with other aspects of the results. Persons seeking to acquire a suitable cat for a child in the family could do well to adopt a calm kitten at weaning, assuring that it has frequent gentle interactions with people of all ages, especially ASD children.

Families are highly motivated to seek out optimal experiences for their ASD children. Most families had a variety of companion animals; thus, the families were increasing their chances of creating a good match for their ASD child. Most cats were supportive of the ASD children, offering them a relationship that often met the lifestyles and needs of the children. The children welcomed affection from the cats that provided love and support in some distinctive ways; not surprisingly, the cats’ affectionate behaviors differ in some ways from those of dogs (24). These findings provide the essential information needed to pursue a controlled prospective clinical study where parents with autistic children could be offered an appropriately reared and socialized pet cat (kitten) of a breed known to be very affectionate, less aggressive, low in fearfulness, playful, and socially outgoing.

## Ethics Statement

Approval for conducting interviews was obtained from the University of California, Davis, Institutional Review Board as Protocols #201018447-1 and #284059-2. The web survey responses were entirely anonymous with an introductory description of informed consent provided to respondents.

## Author Contributions

Conceived and designed all phases of study; collected and analyzed data, and drafted and compiled manuscript: LAH, BLH. APT. Also conceived and designed Phase 1, edited ms: LAL. Provided access to the CHARGE study families with ASD and typically developing children who met eligibility criteria and volunteered for this study; provided confirmed diagnostic and covariate data for the analysis: IHP. Conceived and conducted extended statistical analyses for Phase 1, and drafted text for methods and results pertaining to results and figures that resulted, with specific edits: NHW. Reviewed and edited interim and final draft manuscripts: LAH, APT, NHW, LAL, IH-P, BLH.

## Conflict of Interest Statement

The authors declare that the research was conducted in the absence of any commercial or financial relationships that could be construed as a potential conflict of interest.
